# Development of a bioavailable Hg(II) sensing system based on MerR-regulated visual pigment biosynthesis

**DOI:** 10.1038/s41598-021-92878-6

**Published:** 2021-06-29

**Authors:** Yan Guo, Chang-ye Hui, Lisa Liu, Min-peng Chen, Hong-ying Huang

**Affiliations:** 1 National Key Clinical Specialty of Occupational Diseases, Shenzhen Prevention and Treatment Center for Occupational Diseases, Shenzhen, China; 2 Department of Pathology and Toxicology, Shenzhen Prevention and Treatment Center for Occupational Diseases, Shenzhen, China; 3 Lewis Katz School of Medicine, Temple University, Philadelphia, PA USA

**Keywords:** Environmental biotechnology, Applied microbiology

## Abstract

Engineered microorganisms have proven to be a highly effective and robust tool to specifically detect heavy metals in the environment. In this study, a highly specific pigment-based whole-cell biosensor has been investigated for the detection of bioavailable Hg(II) based on an artificial heavy metal resistance operon. The basic working principle of biosensors is based on the violacein biosynthesis under the control of mercury resistance (*mer*) promoter and mercury resistance regulator (MerR). Engineered biosensor cells have been demonstrated to selectively respond to Hg(II), and the specific response was not influenced by interfering metal ions. The response of violacein could be recognized by the naked eye, and the time required for the maximum response of violacein (5 h) was less than that of enhanced green fluorescence protein (eGFP) (8 h) in the single-signal output constructs. The response of violacein was almost unaffected by the eGFP in a double-promoter controlled dual-signals output construct. However, the response strength of eGFP was significantly decreased in this genetic construct. Exponentially growing violacein-based biosensor detected concentrations as low as 0.39 μM Hg(II) in a colorimetric method, and the linear relationship was observed in the concentration range of 0.78–12.5 μM. Non-growing biosensor cells responded to concentrations as low as 0.006 μM Hg(II) in a colorimetric method and in a Hg(II) containing plate sensitive assay, and the linear relationship was demonstrated in a very narrow concentration range. The developed biosensor was finally validated for the detection of spiked bioavailable Hg(II) in environmental water samples.

## Introduction

Mercury is a naturally occurring heavy metal, and the process of industrialization has resulted in its widespread distribution as an ubiquitous environmental toxin^1^. Methylmercury, as one of the important neurotoxic compounds, is produced with bioavailable mercury as the substrate by environmental anaerobic organisms. Methylmercury becomes bioconcentrated in the food chain, and can cause adverse effects to human health^2^. Monitoring of the bioavailable form of environmental mercury is predictive of the methylation rate of mercury, thereby predicting its biological accumulation in ecosystems. Existing instrumental analysis methods such as atomic absorption spectroscopy, atomic fluorescence spectrometry, and inductively coupled plasma-mass spectrometry are sensitive. However, they are mainly used to determine the total amount of elemental mercury^3^. Whole-cell biosensors, which can even reproduce independently, have the potential to complement currently used physical and chemical analysis methods, allowing a preliminary detection of bioavailable heavy metal ions to assess the impact of them on the environment by simulating environmental microorganisms^4^.

The exceptional sensory and regulatory biological activities of the metalloregulators have made them powerful tools to develop biosensors to cope with prevalent environmental toxins in synthetic biological methods^5,6^. *Escherichia coli* (*E. coli*) has been widely used as the host for whole-cell biosensors, while bioluminescence, fluorescence, and pigmentation (chromogenic substrate) have been the most used output reporters that have been largely documented for the qualitative and quantitative detection of target metals^7^. Although the application of enzymatic reporters usually leads to higher sensitivity, external substrates are always required for the signal output of enzymatic reporters including firefly luciferase, β-galactosidase, and so on^8–10^. A stable fluorescence can be emitted by the fluorescent proteins. However, fluorescence quenching and background fluorescence are always inevitable^11,12^. Thus, novel whole-cell biosensors with stable signal outputs that can be detected conveniently and rapidly are always needed to meet the requirements of practical applications.

Although the traditional enzyme-based colorimetric tests have been widely used in the biosensing of heavy metals due to their unique advantages, an extra incubation is usually necessary for the production of the colorimetric signal, and the detection of output signal relies excessively on equipment^10,13^. A number of visual colored assays based on optimized enzyme-induced biochemical reactions have been successfully used to evaluate the low level of target molecule qualitatively and quantitatively^14,15^. The visual enzyme-free signal-amplification colorimetric immunoassays were demonstrated to facilitate in detecting target molecules with highly sensitivity^16,17^. In addition, visible nanoparticle was successfully used as a novel signal-generation tag to detect small molecules^18^. The development of novel visual colorimetric bioassays is also of great significance for environmental heavy metal monitoring.

With the rapid development of metabolic engineering, some natural pigments, such as the red lycopene, orange β-carotene, red anthocyanin, blue indigoidine, and purple violacein which are the potential clinical therapeutics, have been heterologously biosynthesized in engineered microorganisms successfully^19–23^. Pigmented secondary metabolites have great potential for the development of biosensors that target low-resource areas and countries, since the visual signals can be recognized even without any equipment. The response of whole-cell biosensors with pigments as visible signal outputs have been demonstrated to be recognized by the naked eye, and quantified by the colorimetric methods^24–27^. A genetically engineered red pigment producing *Deinococcus radiodurans* could detect 50 nM to 1 mM Cd (II)^28^. An aromatics degrader *Pseudomonas aeruginosa*, which produces a blue-green pigment, was developed as a bacterial biosensing system for quantification of *N*-butyryl homoserine lactone with a quantitative detection range of 0.11–19.7 μM ^29^. Engineered *Pseudomonas aeruginosa* PAO1 with the pigment pyocyanin as the output signal was demonstrated to respond to 25–1000 nM Hg(II)^27^. The main disadvantage of pigment-based biosensors is that full repression of pigment production when undesired is difficult, as even basal expression of pigment synthase can catalyze the accumulation of visible amounts of pigment. However, this disadvantage can be overcome by a complicated rational design of genetic circuits^20,24^.

These above-mentioned pigments with bright color and high chemical stability have overwhelming advantages as the novel reporters for the detection of heavy metal ions. Violacein, a bisindole with a blue-purple color, is known to have diverse biological activities, including being an antibiotic against Gram-positive pathogens and being an anticancer agent^30,31^. In the present study, bioavailable Hg(II) sensory element originating from natural microorganism was employed to genetically control the biosynthesis of violacein in *E. coli* to develop a fast-responding, minimal-equipment, and colorimetric whole-cell biosensor. The violacein reporter is a liposoluble pigment that exhibits charming blue-purple color which is visual to the naked eye, and measurable absorbance at 490 nm after butanol extraction. The violacein-based biosensor presented in this study independent from extra substrates and specific reagents was demonstrated to be useful in the detection of bioavailable Hg(II) in a sensitive, visual, and stable manner.

## Material and methods

### Bacterial strains, plasmids, and agents

The bacterial strain, vectors and primers used in this study are listed in Table 1. *E. coli* TOP10 was used as the host cell for all the cloning steps and for the assembly of whole-cell biosensors. All engineered bacterial strains were cultured in Luria–Bertani (LB) broth (10 g/L tryptone, 5 g/L yeast extract, and 10 g/L NaCl) supplemented with 50 μg/mL ampicillin. Stock solutions of MnSO_4_, NiSO_4_, CuSO_4_, ZnSO_4_, Pb(NO_3_)_2_, CdCl_2_, and HgCl_2_ were freshly prepared with analytical grade chemicals and distilled water. All oligonucleotide primers were synthesized by Sangon Biotech (Shanghai, China).

**Table 1:** Bacterial strains and plasmids used in this study.

Strains, vectors and primers	Genotypes or description	Origin
**Bacterial stain**		
TOP10	F^-^ Φ80*lac*ZΔM15 Δ*lac*X74 *rec*A1	Invitrogen
**Vectors**		
pET-21a	Amp^R^, T7 promoter, lac operator	Novagen
pET-vio	pET-21a derivative containing the violacien expression cassette (*vioABCDE*) inserted as a *Nde*I/*Sac*I fragment	^22^
pPmer	pET-21a derivative containing *merR* and Pmer divergent promoter region cloned into *Bgl*II and *Xba*I sites	^42^
pPmer-R-Pmer-G	pPmer derivative, an artificial hybrid *mer* operon with transcriptions of *mcherry* and *egfp* under the control of independent Pmer divergent promoter region	^42^
pPmer-vio	pET-vio derivative containing *merR* and Pmer divergent promoter cloned as a *Bgl*II/*Xba*I fragment	This study
pPmer-vio-Pmer-G	pPmer derivative, an artificial hybrid *mer* operon with transcriptions of *vioABCDE* and *egfp* under the control of independent Pmer divergent promoter region	This study
pPmer-G	pPmer derivative carrying promoterless *egfp* cloned into *Nde*I and *Hin*dIII sites	This study
**Primers**		
F-mer	GAAGATCTCTAAGGCATAGCTGACC	This study
R-mer	GCTCTAGAACGTTGGCCCTTTTG	This study
F-Pmer-G	AAGAGCTCATCGCTTGACTCCGTAC	This study
R-Pmer-G	ATAAGAATGCGGCCGCTTATTTGTACAGTTCATCCATAC	This study
F-G	GGAATTCCATATGGTTTCTAAAGGCG	This study
R-G	CCCAAGCTTTTATTTGTACAGTTCATCCATAC	This study

### Plasmids construction

**Figure 1:**
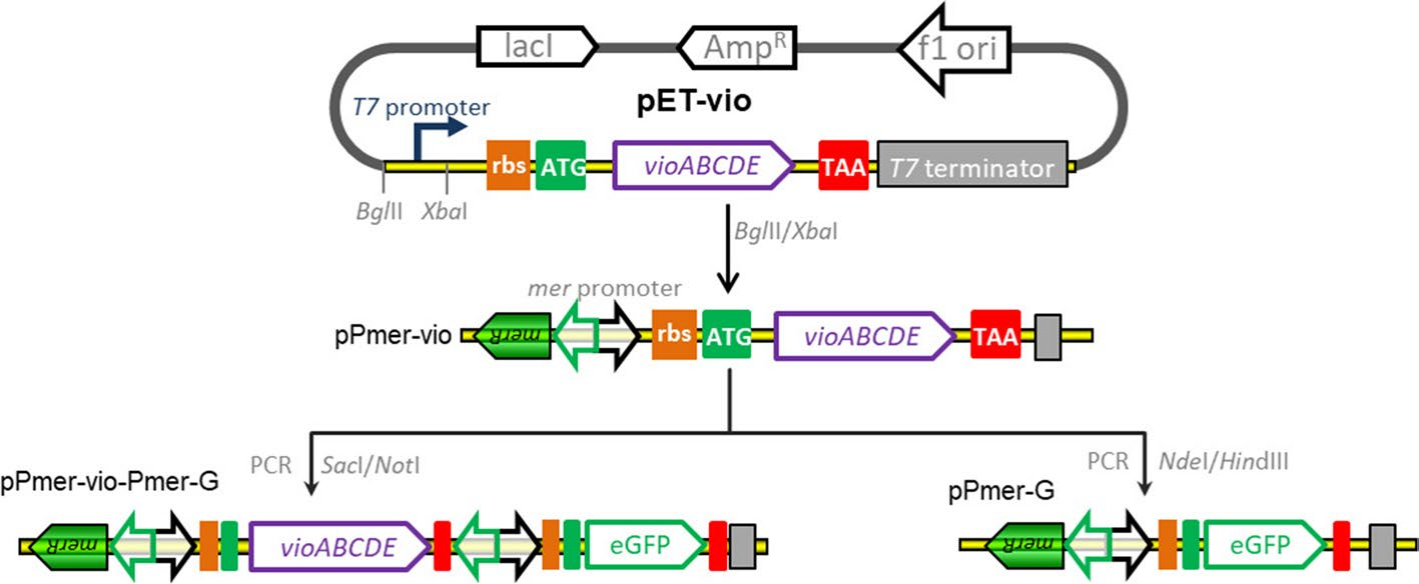
Assembly of artificial *mer* operons for sensing of bioavailable Hg(II). The violacein biosynthesis module and fluorescent reporter module were placed under the control of the *mer* promoter separately or in combination using genetic methods.

The strategy used for the construction of artificial *mer* operons is summarized in Fig. 1. The plasmid pET-vio was previously constructed with a *vioABCDE* gene cluster inserted into the *Nde*I/*Sac*I site of pET-21a. The *Bgl*II-*Xba*I fragment containing the Hg(II) sensing element, the *merR* gene and its divergent *mer* promoter, was amplified from pPmer by PCR using primer pairs F-mer and R-mer. Then, the resultant DNA fragment was cloned into the same sites of pET-vio to generate pPmer-vio, a Hg(II) biosensing construct with a pigment reporter violacein as the output signal. The *Nde*I-*Hind*III fragment containing the sequence encoding eGFP was amplified from pPmer-R-Pmer-G using primer pairs F-G and R-G, and cloned into the same sites of pPmer-vio to produce pPmer-G, a Hg(II) biosensing construct with a fluorescent reporter eGFP as the output signal. The *Sac*I-*Not*I fragment containing an extra *mer* promoter and the open reading frame (ORF) of eGFP was amplified from pPmer-R-Pmer-G using primer pairs F-Pmer-G and R-Pmer-G, and inserted into the same sites of pPmer-vio to yield pPmer-vio-Pmer-G, a Hg(II) biosensing construct with both violacein and eGFP as the output signals. The vectors were all sequenced for verification by Sangon Biotech (Shanghai, China). The DNA sequence of inserted fragments are shown in Fig. S1.

### Detection selectivity assay

*E. coli* TOP10 competent cells were transformed with the plasmid pPmer-vio, spread on the LB plate, and grown overnight at 37 °C. Individual colonies were inoculated in triplicate in 3 mL LB medium, and then cultured overnight at 37 °C. The bacterial culture in stationary phase was inoculated into 3 ml of fresh LB medium (1% inoculum), and grown at 37 °C for 3 h with rotation at 250 rpm to log phase. To evaluate the detection selectivity of violacein-based biosensor, a final concentration of 8 μM Mn(II), Ni(II), Cu(II), Zn(II), Pb(II), Cd(II), or Hg(II) was added to the bacterial culture, followed by incubation at 37 °C for 12 h before cell density and violacein content were analyzed.

To evaluate the influence of various metal ions on the response of violacein-based biosensor toward Hg(II), a mixture of 8 μM Hg(II) with other different metal ions at the concentration of 8 μM was added to the bacterial culture in log phase, followed by incubation at 37 °C for 12 h before cell density and violacein content were analyzed.

### Hg(II) detection with three whole-cell biosensors

The plasmids pPmer-vio, pPmer-G and pPmer-vio-Pmer-G were transformed into *E. coli* TOP10 competent cells. Fresh transformed colonies were inoculated in triplicates in 3 mL LB medium, and then cultured overnight at 37 °C. Overnight cultures (100 μL) were inoculated in 10 mL LB medium, and grown at 37 °C for 3 h until the OD_600_ value reached about 0.4. Then, a final concentration of 8 μM Hg(II) was added to the bacterial culture, followed by a 12-h incubation at 37 °C. Induced cultures were sampled at regular intervals. The bacterial density, the violacein content, and the eGFP fluorescence intensity were then determined.

### Detection sensitivity assay

Fresh transformed TOP10/pPmer-vio colonies were inoculated in triplicates in 3 mL LB medium, and then cultured overnight at 37 °C. Overnight cultures (30 μL) were inoculated in 3 mL LB medium, and grown at 37 °C for 3 h. To evaluate the detection sensitivity of logarithmic phase bacterial cultures toward Hg(II), a final concentration of 0, 0.098, 0.195, 0.39, 0.78, 1.56, 3.125, 6.25, 12.5, 25, 50, 100, or 200 μM Hg(II) was added to the log phase culture, followed by a 5-h incubation at 37 °C before cell density and violacein content were analyzed.

To evaluate the detection sensitivity of lag phase bacterial cultures towards Hg(II), freshly transformed colonies were directly used to inoculate 3 mL of LB medium supplemented with 0, 0.006, 0.012, 0.024, 0.05, 0.098, 0.195, 0.39, 0.78, 1.56, 3.125, 6.25, or 12.5 μM Hg(II). This was then followed by a 12-h incubation at 37 °C before cell density and violacein content were analyzed.

### Plate sensitivity assay

Recombinant TOP10/pPmer-vio was cultured in LB medium overnight at 37 °C. The bacterial culture in stationary phase was inoculated into fresh LB medium (1% inoculum), and grown at 37 °C for 3 h to reach log phase. 50 μL exponential-phase culture was spread on LB agar in a 6-well culture plate supplemented with 0, 0.006, 0.012, 0.024, 0.05, 0.098, 0.195, 0.39, 0.78, 1.56, 3.125, or 6.25 μM Hg(II), followed by culture at 37 °C overnight before the results were observed.

### Detection of environmental water samples by the developed biosensor

Developed violacein-based biosensor was applied to different water samples including distilled water, tap water, and lake water collected from a local lake as described previously^22,27^. In one group, 2.7 mL of exponential phase LB-cultures of TOP10/pPmer-vio were mixed with either purified water, tap water or lake water in 300 μL aliquots, and the mixtures were spiked with 0, 3.125, 6.25, or 12.5 μM Hg(II), respectively. In another group, 2.7 mL of lag phase LB-cultures of TOP10/pPmer-vio were mixed with three kinds of water samples in 300 μL aliquots, and the mixtures were spiked with 0, 0.006, 0.012, or 0.024 μM Hg(II), respectively. The cultures continued to be cultivated for 12 h at 37 °C before bacterial density and violacein content were determined.

### Determination of the reporter signals

Violacein was extracted and quantified as described previously^22,32^. Bacterial cells in 1 mL culture were harvested by centrifugation at 5000 g for 5 min, resuspended in 200 μL of lysis solution (2% SDS in distilled H_2_O), and vortexed violently for 10 min. The released violacein from cellular cytoplasm was then extracted by adding 400 μL butanol, followed by vortexing for 10 min. The butanol phase (upper) was obtained by centrifugation at 8000 g for 2 min. Aliquots of 150 μL were read at 490 nm using a microplate reader (Bio-Rad, USA).

The fluorescence intensity of eGFP expressed in bacterial cytoplasm was determined with Lumina fluorescence spectrometer (Thermo, USA) as described previously^11,33^. A 3-mL aliquot of bacterial culture or diluent was added in a 1-cm path low fluorescence background quartz cuvette. The samples were excited at 488 nm, and the fluorescence emission was recorded at 507 nm.

## Results

### Assembly of pigment-based Hg(II) whole-cell biosensor

**Figure 2:**
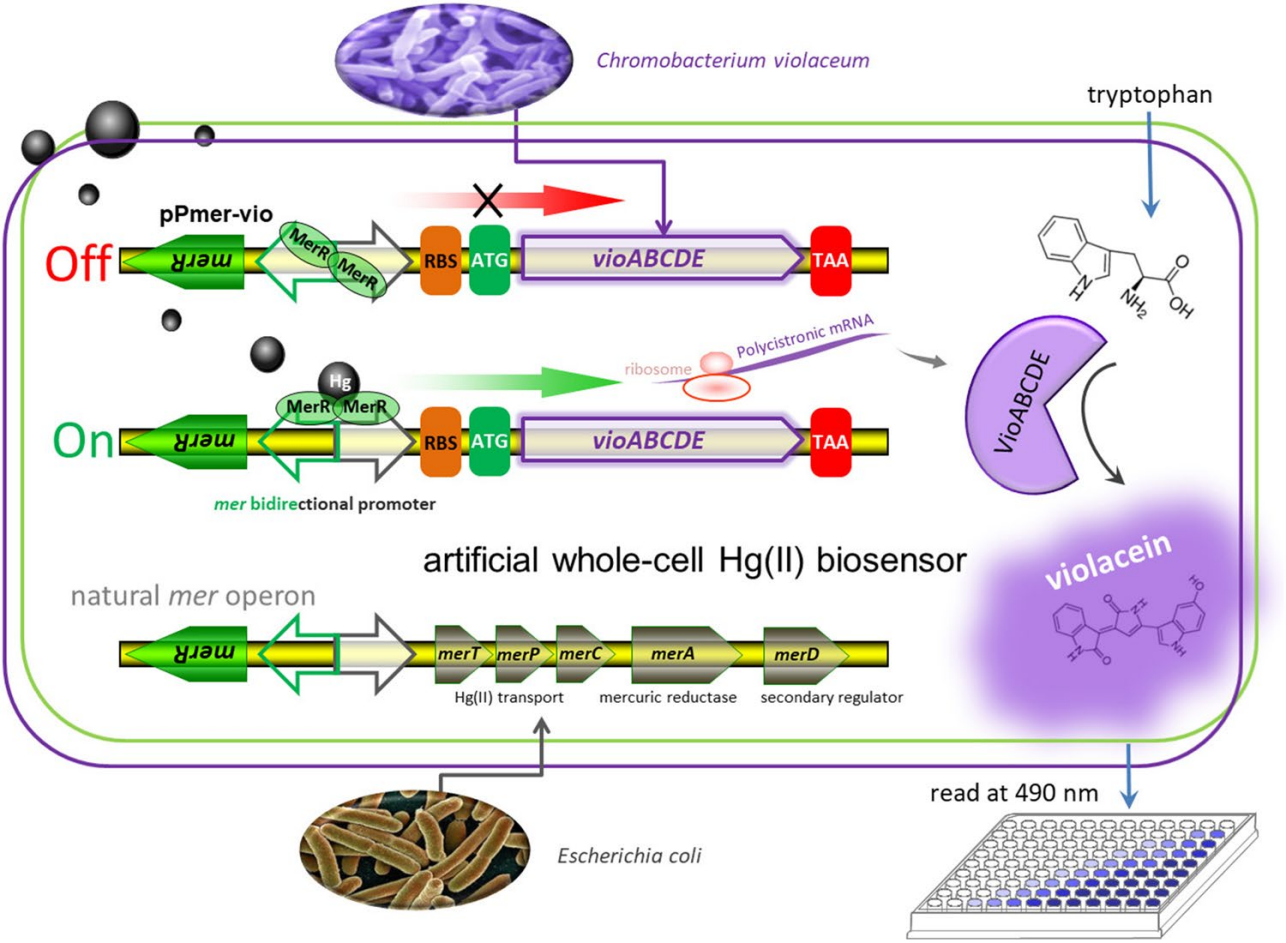
Engineered Hg(II) whole-cell biosensor based on MerR-regulated visual violacein production. The archetype of natural *mer* operon originated from *E. coli* is shown at the bottom of the figure. To assemble Hg(II)-responsive visual biosensor, the mercury detoxification gene cluster *merTPCAD* was substituted with the violacein biosynthetic gene cluster *vioABCDE*. The violacein biosynthetic module, originated from *Chromobacterium violaceum*, is transcriptionally regulated by the Hg(II) sensing element to enable a whole-cell violacein-based Hg(II) biosensor. The violacein, produced with bioavailable Hg(II) induction, can be extracted with organic solvents such as butanol, and quantified by a microplate reader at 490 nm.

The *mer* operon, located on transposon Tn21 from *E. coli*, is one of the best characterized bacterial mercury resistance operons^34^. Binding of Hg(II) to dimeric MerR leads to DNA distortion and transcriptional activation of the downstream Hg(II) detoxification gene cluster (*merTPCAD*)^35^. In this study, the *merTPCAD* gene cluster was substituted with the violacein biosynthetic gene cluster (*vioABCDE*) originated from *Chromobacterium violaceum*, which is under the control of the *mer* divergent promoter (Fig. 2). The metalloregulatory MerR is a Hg(II)-responsive transcriptional repressor and activator. Apo MerR dimer binds to the divergent *mer* promoter as a repressor to block transcription initiation of the downstream *vioABCDE* gene cluster. However, this dimeric MerR is converted into an activator upon Hg(II) binding, and dimeric MerR associated with bioavailable Hg(II) can specifically activate transcription of a polycistronic gene cluster composed of *vioA*, *vioB*, *vioC*, *vioD*, and *vioE*. Then, the Hg(II) inducible five violacein biosynthetic enzymes catalyze the condensation of two molecules of endogenous L-tryptophan to produce violacein in the following pathway: VioA-VioB-VioE-VioD-VioC^36^. Previous studies have demonstrated that intracellular violacein could be extracted with organic solvents following cell lysis, and quantified using a colorimetric method at 490 nm^22^.

### Evaluation of the detection specificity of the violacein-based biosensor

**Figure 3:**
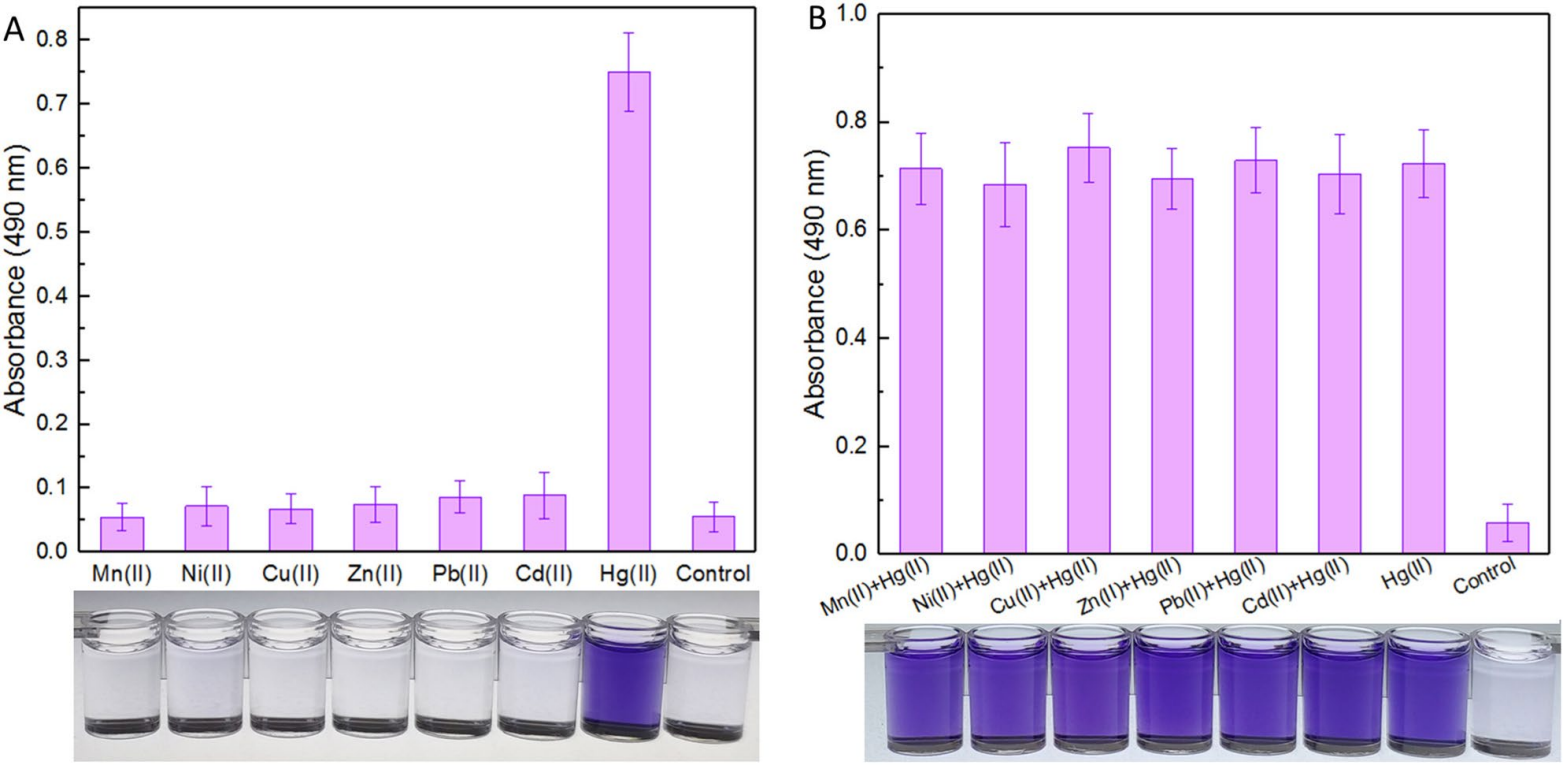
The biodetection selectivity of engineered whole-cell Hg(II) biosensor. Biosensor cells in the exponential growth phase were exposed to 8 μM of various metal ions alone (**A**) or in combination (**B**) at 37 °C for 12 h. The control groups were not supplemented with metal ions. The butanol phases containing violacein were prepared and read at 490 nm in a microplate reader. The photo shown at the bottom is representative of three independent experiments with similar results. The absorbance values of pigment were normalized to bacterial cell density at 600 nm. The results are shown as the mean of three independent assays ± the standard deviation.

The response of recombinant TOP10/pPmer-vio toward various metal ions was detected first. Exponential-phase engineered bacteria were cultured in the presence of 8 μM of various metal ions. The bacterial cells were harvested after a 12-h induction, and the accumulated violacein was extracted and quantified. As shown in Fig. 3A, engineered TOP10/pPmer-vio specifically responded to Hg(II), but responded silently to other different metal ions. The blue-purple color in the butanol phase with Hg(II) exposure could be clearly recognized by the naked eye.

To investigate whether other metal ions exert any influences on the performance of violacein-based biosensor toward Hg(II) or not, exponential-phase engineered bacteria were cultured in the presence of 8 μM Hg(II) accompanied with other interfering metal ions. As shown in Fig. 3B, all the bacterial cultures exposed with Hg(II) alone or in combination, displayed the obvious accumulation of violacein. The presence of other metal ions exerted a slight influence on the performance of the Hg(II)-specific biosensor developed in this study.

### Comparison of the performance of whole-cell biosensors in response to Hg(II)

In order to compare the performance of the novel pigment reporter violacein and the widely used fluorescent reporter eGFP, both single fluorescent signal output genetic construct pPmer-G and double signals output genetic construct pPmer-vio-Pmer-G were assembled.

**Figure 4:**
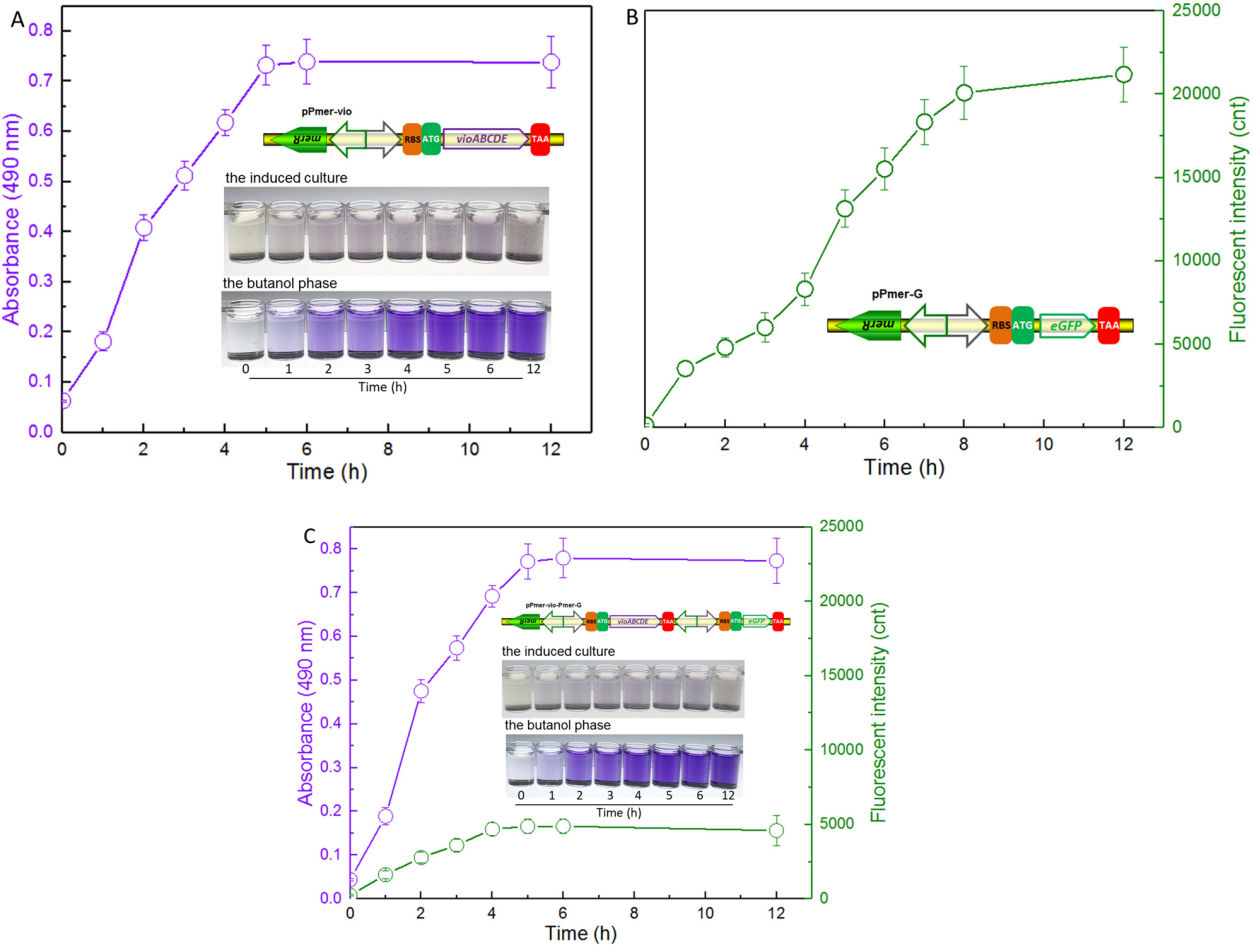
Time courses of reporter signals generated by three whole-cell biosensors with 8 μM Hg(II) exposure. Quantitative time-course profiles of whole-cell biosensors TOP10/pPmer-vio (**A**), TOP10/pPmer-G (**B**), and TOP10/pPmer-vio-Pmer-G (**C**) to Hg(II). Three kinds of bacterial cells in exponential growth phase were exposed to 8 μM Hg(II) at 37 ^o^C. The fluorescent signal and pigment-based color change were detected at regular time intervals. Both of the reporter signals were normalized to bacterial cell density at 600 nm. The results are shown as the mean of three independent assays ± the standard deviation.

Exponential-phase biosensor cells were cultured in the presence of 8 μM Hg(II). Under the exposure of Hg(II), engineered TOP10/pPmer-vio constantly produced violacein within the first 5 h of induction onset. Although the water-insoluble violacein was mostly accumulated in the cytoplasm, the color change of induced cultures could still be distinguished at 1 h. Importantly, the color change of the butanol extraction phase was significantly more obvious than that of the bacterial culture (Fig. 4A).

As shown in Fig. 4B, the expression of eGFP in engineered TOP10/pPmer-G continuously increased with Hg(II) exposure. The fluorescent signal output did not rise up after 8 h of induction, and it took two more hours than violacein-based biosensor to get the maximum reporter signal under the same experimental conditions. To further compare the performance of two kinds of reporters in one genetic construct, a double-signal output construct pPmer-vio-Pmer-G was assembled. It allows both violacein biosynthetic gene cluster and eGFP encoding gene to be transcribed under the control of its own independent *mer* promoter (Fig. S2). The result is shown in Fig. 4C. The performance of reporter violacein was almost not influenced by simultaneous co-production of the eGFP reporter. The maximum violacein signal output was still obtained after a 5-h induction. More importantly, the signal intensity of violacein in a double-signal biosensing construct (Fig. 4C) was similar to that in the single-signal biosensing construct pPmer-vio (Fig. 4A). Although the maximum signal output of eGFP was obtained after a 4-h induction, the fluorescent intensity in the double-signal biosensing construct (Fig. 4C) was just a quarter of that in the single fluorescent signal construct (Fig. 4B).

### Evaluation of the detection sensitivity of the violacein-based biosensor in response to Hg(II)

**Figure 5:**
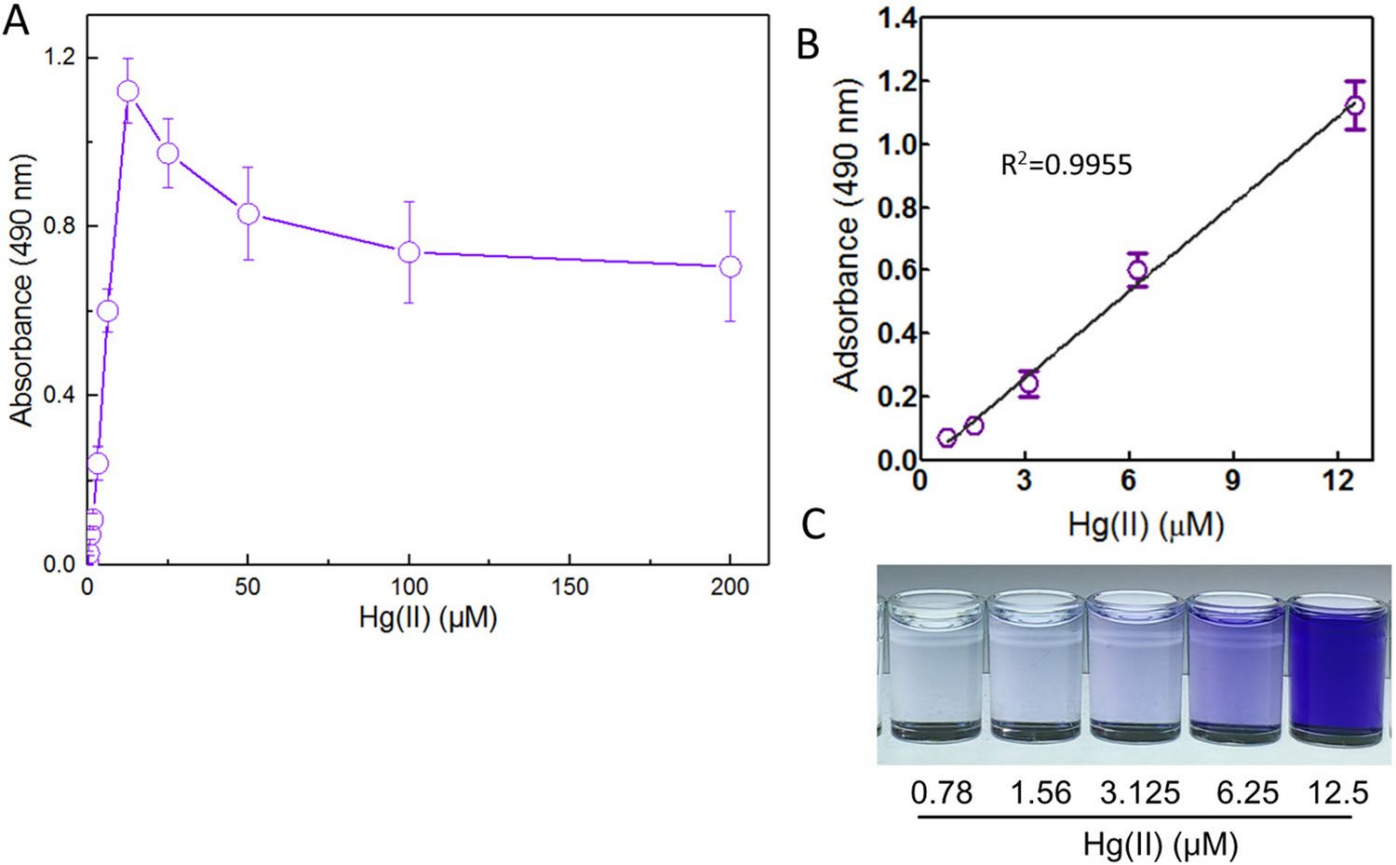
The response of exponential-phase culture of TOP10/pPmer-vio induced with increased concentrations of Hg(II). Exponential-phase culture of TOP10/pPmer-vio was induced with increased concentration of Hg(II) at 37 °C for 5 h. Whole-cell biosensor dose–response (**A**) and linear response (**B**) to Hg(II). The butanol phases containing violacein with 0.78–12.5 μM Hg(II) induction (**C**). Shown is one representative of three independent experiments with similar results. The background intensity (the absorbance at 490 nm with no Hg(II) exposure) was subtracted from each reading. The results are shown as the mean of three independent assays ± the standard deviation. The linear relationship was in the concentration range of 0.78–12.5 μM (R^2^ = 0.9955).

Firstly, exponentially growing biosensor cells were treated with 0, 0.098, 0.195, 0.39, 0.78, 1.56, 3.125, 6.25, 12.5, 25, 50, 100, and 200 μM Hg(II) at 37 °C. After a 5-h induction, the accumulated violacein was extracted and quantified. As shown in Fig. 5A, the biosensor cells could respond to concentrations as low as 0.39 μM Hg(II) in a colorimetric method (absorbance at 490 nm = 0.028 ± 0.006), and the production of violacein was significantly increased in biosensor cells exposed to 0.39–12.5 μM Hg(II). The production of violacein showed a downward trend with higher than 12.5 μM Hg(II) exposure owing to the cytotoxicity of mercury. A good linear relation between pigment response and the concentration of Hg(II) was in the concentration range of 0.78–12.5 μM (Fig. 5B). The color change of the butanol phase was clearly distinguished with the naked eye under 6.25 μM Hg(II) induction (Fig. 5C).

**Figure 6:**
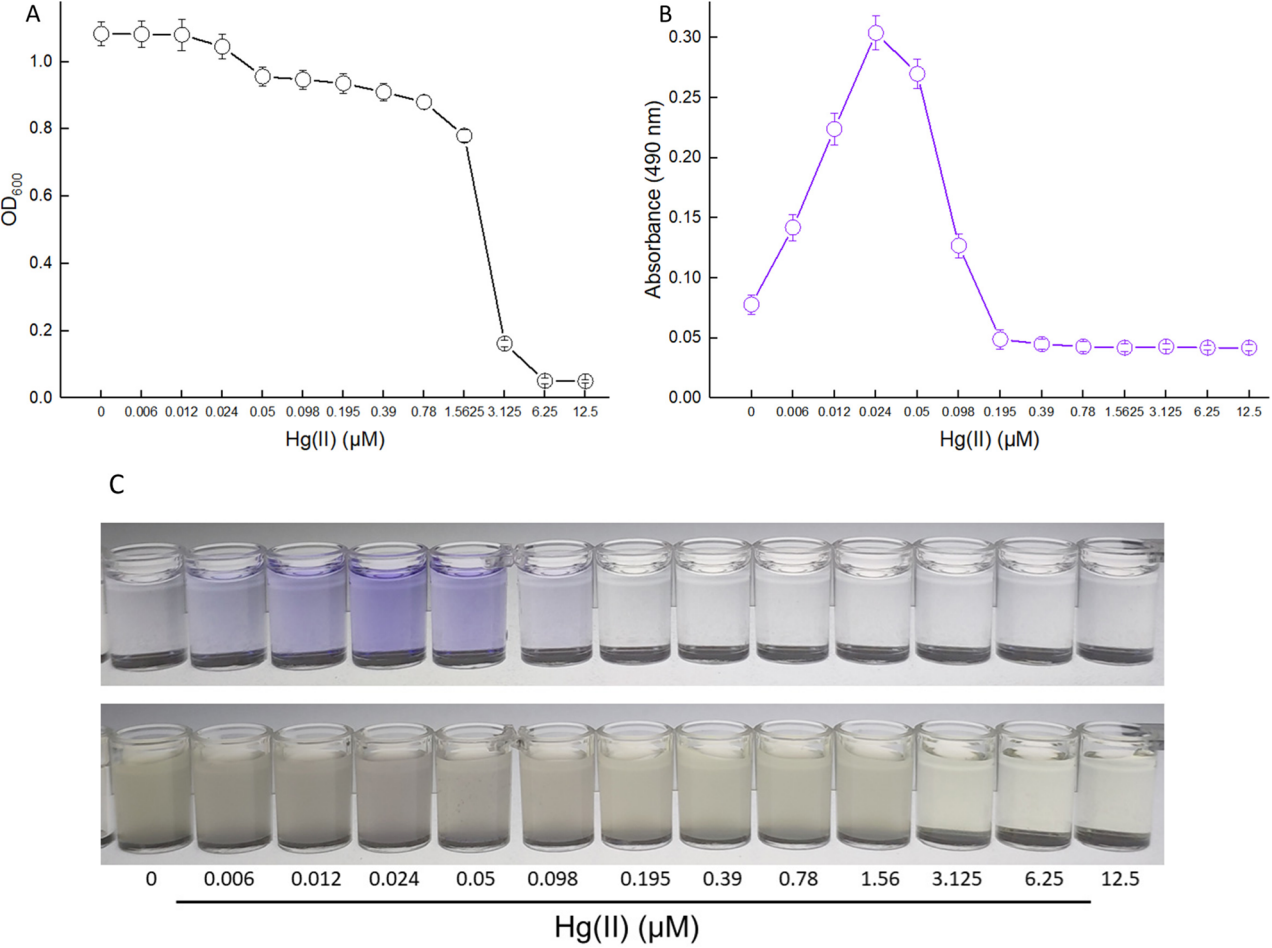
The response of resting TOP10/pPmer-vio induced with increased concentrations of Hg(II). *E. coli* TOP10 harboring the plasmid pPmer-vio was inoculated in LB broth containing increased concentrations of Hg(II) at 37 °C for 12 h. The bacterial density (OD_600_) was determined (**A**), and then the violacein was extracted and quantified at 490 nm (**B**). The induced culture and the butanol phase with increased concentrations of Hg(II) exposure (**C**). Shown is one representative of three independent experiments with similar results.

Secondly, non-growing (lag phase) biosensor cells were exposed to 0–12.5 μM Hg(II) at 37 °C for 12 h. Then, the bacterial density and violacein content were determined. As shown in Fig. 6A, the growth of biosensor cells was slightly decreased with less than 1.56 μM Hg(II) exposure. Due to obvious cytotoxicity, biosensor cells could not reproduce at above 3.125 μM Hg(II). The levels of pigment reporter increased significantly with an increased concentration of Hg(II) exposure, and the maximum response of violacein was obtained at 0.024 μM Hg(II) (Fig. 6B). A linear relation between the absorbance at 490 nm and the concentration of Hg(II) was in the concentration range of 0–0.012 μM (R^2^ = 0.9950, data not shown). Although the growth of biosensor cells did not obviously decrease with 0.05–0.195 μM Hg(II) exposure, the response of violacein dropped sharply within this exposure concentration range of Hg(II). The biosensor cells responded silently at 0.195 μM Hg(II) and above (Fig. 6B). The color change of induced cultures was not obvious. However, the color change of the butanol phases could be distinguished with the naked eye within 0.006–0.098 μM Hg(II), and the strongest response of violacein with 0.024 μM Hg(II) exposure could be recognized (Fig. 6C).

**Figure 7:**
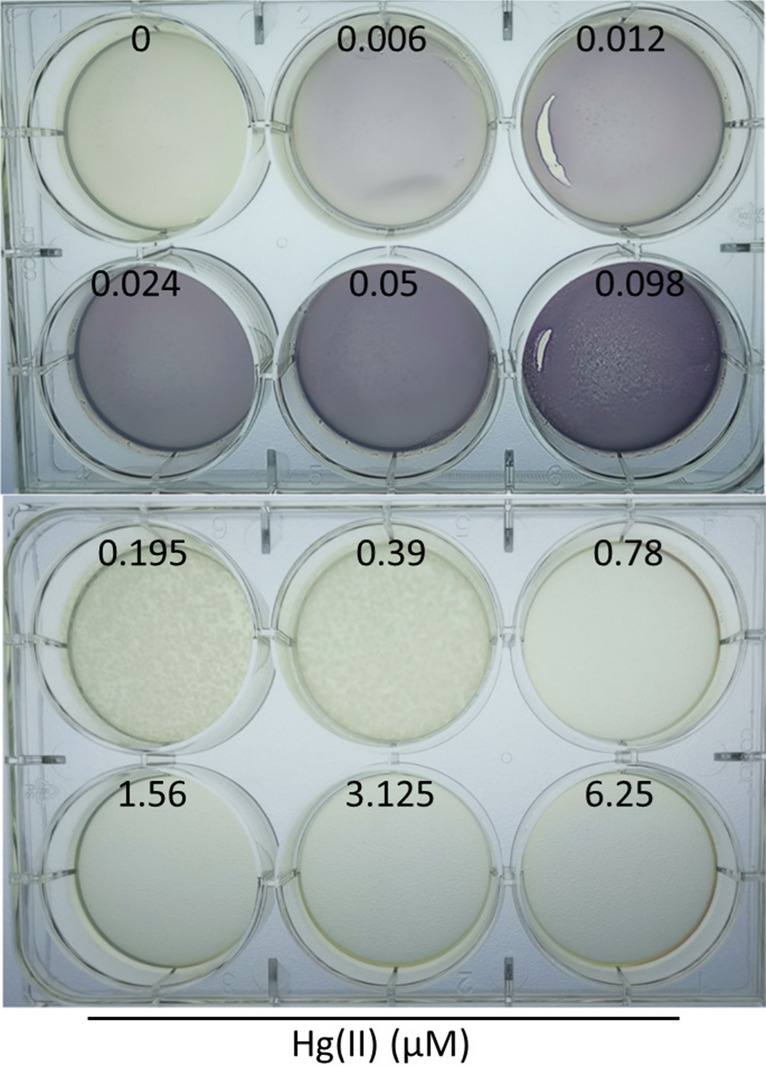
The detection sensitivity of TOP10/pPmer-vio toward Hg(II) on the LB-agar plate. Exponential-phase culture of TOP10/pPmer-vio was spread on LB-agar plates with increasing concentrations of Hg(II), and cultured at 37 °C overnight. A representative image of three independent experiments with similar results is shown.

The response of biosensor cells to Hg(II) on LB agar plate was also determined. As shown in Fig. 7, the exponentially growing biosensor cells were spread on the LB agar and grown at 37 °C overnight. The biosensor cells incubated on the agar plate responded to as low as 0.006 μM Hg(II). Significantly increased pigment accumulation was observed in the concentration range of 0.006–0.098 μM Hg(II). Although there was bacterial lawn grown at 0.195 and 0.39 μM Hg(II), no production of violacein was observed. In addition, no obvious bacterial lawn on the agar surface was observed at 0.78 μM Hg(II) and above owing to the obvious cytotoxicity of Hg(II).

### Performance of the violacein-based biosensor treated with environmental water samples

To validate the ability of the violacein-based biosensor to detect bioavailable Hg(II) in water samples from different sources, biosensor cells in the exponential phase were exposed to 0, 3.125, 6.25, and 12.5 μM Hg(II) dispersed in culture mixture based on either purified water, tap water or lake water in one group, and biosensor cells in the lag phase were exposed to 0, 0.006, 0.012, and 0.024 μM Hg(II) dispersed in culture mixture based on different water samples in another group. It was noted that an increase in the violacein content (the absorbance at 490 nm) correlated with an increase in Hg(II) concentration among the two groups after overnight culture, and the bacterial density (the OD_600_ value) slightly decreased due to the cytotoxicity of high concentrations of Hg(II). Importantly, no significant difference (*P* > 0.05) was observed when comparing the violacein contents in the butanol phase with the same concentration of Hg(II) exposure in either purified water, tap water or lake water.

## Discussion

One of the best characterized bacterial mercury detoxification operons is the *mer* operon that confers bacterial resistance to inorganic Hg(II)^37^. Dimeric MerR is a metalloregulatory transcriptional switch, converting from repressor to activator when associated with bioavailable mercury (Fig. 2). A substantial number of whole-cell biosensors have been developed using the protein MerR as the sensing element. As shown in Table. 2, all the previously reported MerR-based biosensors specifically responded to Hg(II) due to the extremely high selectivity of metalloregulator MerR toward its cognate Hg(II). Both laboratory *E. coli* strains and environmental bacterial strains (No. 7–10 in Table 2) were used as the hosts for harboring the biosensing genetic constructs. Furthermore, a few whole-cell biosensors with chromosomally integrated sensing modules were developed for a more stable biosensing. Compared with most plasmid-based biosensors, the detecting sensitivities of chromosomally based biosensors were not decreased due to a decreased copy number of the biosensing cassette^33,38,39^.

**Table 2:** Comparison of developed whole-cell biosensors for the detection of bioavailable Hg(II).

No	Host cells	Biosensing modules	growth phase when induction	Detection range	LOD (μM)	Induction time	Specificity	Ref
1	*E. coli* MT102-PIR	*merR*-Pmer-*luxCDABE*	lag phase	–	0.0012	80 min	Hg(II)	^13^
		*merR*-Pmer-*lacZ*	lag phase	–	0.0024	4 h	Hg(II)	
		*merR*-Pmer-*gfp*	lag phase	–	0.5	16 h	Hg(II)	
2	*E. coli* DH5α	*zntR*-Pznt-*egfp*-*hj1*	lag phase	2.5–7.5	1.0	6 h	Cd(II), Hg(II)	^50^
3	*E. coli* JM109	chromosomally based *merR*-Pmer-*gfp*	lag phase	0.1–1.7	–	12 h	Hg(II)	^38^
4	*E. coli* TOP10	*merR*-Pmer-*mcherry*	exponential phase	6.25–200	–	8 h	Hg(II)	^42^
5	*E. coli* DH5α	*merR*-Pmer-*rfp*	Late exponential phase	0.05–10	–	2 h	Hg(II)	^51^
6	*E. coli* DH5α	*mer*-*rfp* quorum-sensing system	exponential phase	0.01–0.25	0.01	6 h	Hg(II)	^46^
7	*Sphingobium* SA2	chromosomally based partial *merA*-*gfp*	lag phase	0.02–0.04	-	5 h	Hg(II)	^39^
8	*Pseudomonas putida*	chromosomally based *merR*-Pmer-*egfp*	lag phase	0.2–1.4	–	16 h	Hg(II)	^33^
9	*Pseudomonas aeruginosa*	*merR*-Pmer-*phzM*-Pmer-*phzS*	early-lag phase	0.025–1	0.01	12 h	Hg(II)	^27^
10	*Enterobacter cloacae*	*merR*-Pmer-*lux*	early exponential phase	0.002–7.98	0.001	1 h	Hg(II)	^52^
11	*E. coli* TOP10	*merR*-Pmer-*vioABCDE*	exponential phase	0.78–12.5	0.39	5 h	Hg(II)	This study
			lag phase	–	0.006	12 h	Hg(II)	

LOD: limit of detection, lux: luciferase, lacZ: β-galactosidase, gfp: green fluorescent protein, zntR: Zn(II)-responsive transcriptional regulator, rfp: red fluorescent protein, merA: mercuric reductase, phzM: phenazine-specific methyltransferase, phzS: FAD-dependent monooxygenase.

The natural violacein synthetic pathway was employed in the present study to assemble a colorimetric whole-cell biosensor toward Hg(II). The selectivity of this biosensor was then investigated. Like most previously reported biosensors with MerR as the sensing element (Table 2), the developed violacein-based biosensor specifically responded to Hg(II), and the selectivity of response was not influenced by various other metal ions (Fig. 3). The specificity of this biosensor with violacein as the output signal was demonstrated to depend entirely on the extremely high specificity of MerR toward Hg(II).

An overnight induction was usually used to obtain the maximum fluorescent signal output when induced at the lag phase of biosensor cells, and > 6 h induction was used when induced at the exponential phase (Table 2). The response characteristics of the fluorescent reporter eGFP and the violacein biosynthetic module under the control of the same Hg(II) sensory element were subsequently compared. Acquisition of stable green fluorescent signal required longer incubation (above 8 h) than acquisition of stable pigment signal (5 h) under the same culture conditions (Fig. 4). To further compare the response ability of two reporters, both the eGFP reporter and the violacein reporter were placed downstream of its independent *mer* promoter in one genetic construct. Then, the performance of violacein reporter was demonstrated to be significantly better than that of the eGFP reporter (Fig. 4C). The response of violacein was not changed with the introduction of extra eGFP. However, the response of eGFP was significantly decreased probably due to the cytotoxicity of overproduced violacein in the cytoplasm^22^. The results of this comparative assay show that the production of violacein reporter has obvious advantages over the expression of traditional fluorescence protein reporter in either response time or response efficiency.

The developed violacein-based biosensor responded in a quantitative manner to Hg(II) within a certain concentration range. It appears that induction of non-growing (lag phase) biosensor cells (Fig. 6) was far more sensitive than induction of exponentially growing biosensor cells (Fig. 5). Previous studies have demonstrated that both high cell densities and complex medium decrease the amount of intracellular bioavailable Hg(II)^33,40–42^. Thus, it is not difficult to see why exponentially growing biosensor cells (OD_600_ = 0.4) responded to higher concentration of Hg(II) than non-growing biosensor cells (OD_600_ = 0.01). The metabolism of biosensor cells in log phase is much more vigorous than biosensor cells in lag phase. Although exponential-phase culture of biosensor only responded to 0.39 μM Hg(II) and above, biosensor cells in this phase could resist the cytotoxicity of high concentration of Hg(II). The bacterial cells still survived with 200 μM Hg(II) exposure, and violacein was produced at a certain measurable level (Fig. 5). A good linear relationship was observed in a high concentration range of 0.78–12.5 μM in exponential-phase culture. In fact, lag-phase culture of biosensor could respond to concentrations as low as 0.006 μM Hg(II), and a linear relationship was observed in a very narrow and very low concentration range between 0 and 0.012 μM (Fig. 6). This finding is consistent with previous studies suggesting that induction of biosensor cells in a vigorous growth state is important to get a response within a wide and high Hg(II) concentration range (Table. 2). In addition, the highest absorbance at 490 nm in lag-phase culture (0.304 ± 0.014, at 0.024 μM) was significantly lower than that in exponential-phase culture (1.122 ± 0.076, at 12.5 μM). Thus, it can be seen that high violacein signal output depends on the growth stage of biosensors at the time of induction. A substantial number of studies have also demonstrated that high recombinant protein expression was usually obtained by adding inducer during the log growth phase^11,33,43,44^.

Whole-cell biosensors were previously demonstrated to respond to low concentrations of Hg(II) when the bacterial cell density is low. Lag-phase culture of green fluorescence protein (GFP)-based biosensor with MerR as a sensory element could respond to concentrations as low as 0.5 μM Hg(II)^13^. However, exponential-phase culture of eGFP-based biosensor only responded to 3.125 μM Hg(II) and above^42^. The difference between these tests may be a result of the differences in assay conditions. Rational design of genetic circuit is another key factor for improved performance of biosensors except for the optimization of detection conditions. The expression level of sensor MerR was used as a regulator in the genetic circuit for tuning detection sensitivity, and the detection limit of exponential-phase culture of biosensor with GFP as an output signal was enhanced to 0.1 μM Hg(II)^45^. A novel Hg(II) biosensor, which could detect concentrations as low as 0.02 μM Hg(II), was developed by combining quorum sensing-based positive feedback systems. Both the sensitivity and the fluorescent intensity of engineered bacterial cells were greatly improved^46^. Exponential-phase culture of violacein-based biosensor could still respond to 0.39 μM Hg(II) and above, and it shows a certain advantage over the traditional fluorescent reporter. Among previously reported biosensors, the highest detection sensitivity was observed in luciferase-based biosensors. However, the lag phase culture of violacein-based biosensor could detect Hg(II) at similar concentrations (Table 2). The United States Environmental Protection Agency (USEPA) recommends that the criteria maximum concentration of mercury in freshwater is 1.4 μg/L (0.007 μM)^47^. The developed violacein-based whole-cell biosensor in lag phase could detect below the USEPA criteria of maximum concentration to prevent acute toxicity in aquatic organisms.

Compared with other pigment-based reporters^28,29,48^, violacein has an easily recognizable blue-purple color, and the color change in the butanol phase could be distinguished even when biosensors were exposed to a low concentration of Hg(II). In addition, the pigment violacein possesses unique properties such as excellent thermal stability, and chemical inertness^31^. The violacein signal is very stable and unchanged after long-term storage of samples (data not shown). Water-insoluble violacein was easy to agglutinate in the cytoplasm. An extra cell lysis and butanol extraction with a long-term violent vortex were needed before a colorimetric test^22,49^. These extra procedures can lead to increase test time and cost. In addition, the growth phase of induced biosensor cells was demonstrated to be a key factor that influenced the reproducibility of the violacein-based biosensor. As shown in Fig. S3, TOP10/pPmer-vio in different growth phases was exposed to increased concentrations of Hg(II). Early exponential-phase cultures could respond to the lowest concentration of Hg(II) at 0.098 μM, but could not resist the cytotoxicity of concentrations above 6.25 μM Hg(II). Responses of late exponential-phase cultures were also decreased due to decreased cell metabolic activity. Importantly, the performances of three independent repeats in the same growth phase were relatively consistent. The above results revealed that the stable reproducibility was largely determined by the growth phase of biosensor cells (the bacterial density) at the beginning of induction.

**Figure 8:**
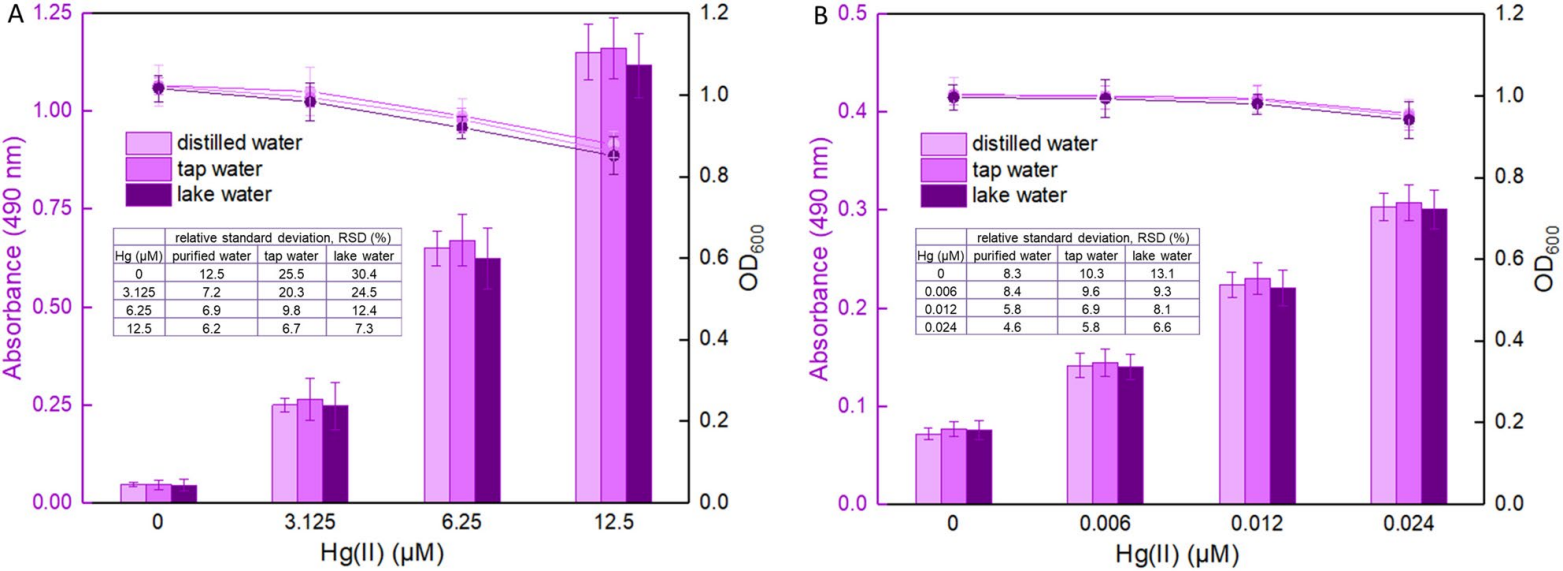
Performance of biosensor cells against environmental water samples spiked with different concentrations of Hg(II). LB-incubated exponential-phase cultures (**A**) or LB-incubated lag-phase cultures (**B**) of whole-cell biosensor TOP10/pPmer-vio were exposed to varying concentrations of Hg(II) in the following water samples: sterile distilled water, unsterilized tap water and unsterilized lake water. After culturing at 37 °C for 12 h, the bacterial density (OD_600_) was first determined (point line diagram, right-Y scale), and then the butanol-extracted violacein was determined at 490 nm (bar chart, left-Y scale). Relative standard deviation (RSD) is shown in the table embodied in the figure.

Environmental monitoring and accurate risk assessment of mercury should be performed taking into account the bioavailability of mercury. It is well known that enormous microorganisms and significant amounts of contaminants including mercury exist in the environmental water. An ampicillin resistance gene is located in the violacein-based biosensing construct, and the reporter violacein displays activity against different Gram-positive bacteria^30^. Both antibacterial properties can ensure the biosensor cells survive amongst the contaminable environmental bacteria. Our preliminary studies of the violacein-based biosensor have shown potential to measure spiked bioavailable Hg(II) in environmental water samples (Fig. 8). The RSD values were greatly decreased with increased concentrations of spiked Hg(II). This suggests that the influence of unknown impurities originating from environmental water was gradually weakened when the concentration of spiked Hg(II) was increased. The stability and accuracy of Hg(II) quantitative detection should be enhanced with increased concentrations of Hg(II) in aqueous environmental samples. In summary, these findings suggest that the violacein-based biosensor has potential for ecotoxicological assessment of mercury-polluted environmental water samples.

## Supplementary Information

Supplementary Information.

## References

[1] Bernhoft, R.A. Mercury toxicity and treatment: a review of the literature. *J Environ Public Health* **2012**, 460508 (2012). 10.1155/2012/460508 PMC3253456 22235210

[2] Regnell O, Watras CJ (2019). Microbial mercury methylation in aquatic environments: a critical review of published field and laboratory studies. Environ. Sci. Technol..

[3] Suvarapu LN, Baek SO (2017). Recent studies on the speciation and determination of mercury in different environmental matrices using various analytical techniques. Int. J. Anal. Chem..

[4] Bereza-Malcolm LT, Mann G, Franks AE (2015). Environmental sensing of heavy metals through whole cell microbial biosensors: a synthetic biology approach. ACS Synth. Biol..

[5] Jung J, Lee SJ (2019). Biochemical and biodiversity insights into heavy metal ion-responsive transcription regulators for synthetic biological heavy metal sensors. J. Microbiol. Biotechnol..

[6] Guo Y (2021). Development of cadmium multiple-signal biosensing and bioadsorption systems based on artificial *cad* operons. Front Bioeng Biotechnol.

[7] Kim HJ, Jeong H, Lee SJ (2018). Synthetic biology for microbial heavy metal biosensors. Anal. Bioanal. Chem..

[8] Guo Y, Hui CY, Liu L, Zheng HQ, Wu HM (2019). Improved monitoring of low-level transcription in *Escherichia coli *by a beta-galactosidase alpha-complementation system. Front Microbiol..

[9] Smirnova DV, Ugarova NN (2017). Firefly luciferase-based fusion proteins and their applications in bioanalysis. Photochem. Photobiol..

[10] Hui CY (2020). Construction of a RFP-lacZα bicistronic reporter system and its application in lead biosensin. PLoS ONE.

[11] Hui CY, Guo Y, Zhang W, Huang XQ (2018). Rapid monitoring of the target protein expression with a fluorescent signal based on a dicistronic construct in *Escherichia coli*. AMB Express.

[12] Gautier A, Tebo AG (2018). Fluorogenic protein-based strategies for detection, actuation, and sensing. BioEssays.

[13] Hansen LH, Sorensen SJ (2000). Versatile biosensor vectors for detection and quantification of mercury. FEMS Microbiol. Lett..

[14] Ren R, Cai G, Yu Z, Tang D (2018). Glucose-loaded liposomes for amplified colorimetric immunoassay of streptomycin based on enzyme-induced iron(II) chelation reaction with phenanthroline. Sensor Actuat. B-Chem..

[15] Lai W, Wei Q, Xu M, Zhuang J, Tang D (2017). Enzyme-controlled dissolution of MnO_2_ nanoflakes with enzyme cascade amplification for colorimetric immunoassay. Biosens. Bioelectron..

[16] Ren R, Cai G, Yu Z, Zeng Y, Tang D (2018). Metal-polydopamine framework: an innovative signal-generation tag for colorimetric immunoassay. Anal. Chem..

[17] Gao Z, Lv S, Xu M, Tang D (2017). High-index {*hk*0} faceted platinum concave nanocubes with enhanced peroxidase-like activity for an ultrasensitive colorimetric immunoassay of the human prostate-specific antigen. Analyst.

[18] Gao Z, Qiu Z, Lu M, Shu J, Tang D (2017). Hybridization chain reaction-based colorimetric aptasensor of adenosine 5'-triphosphate on unmodified gold nanoparticles and two label-free hairpin probes. Biosens. Bioelectron..

[19] Zhang W, Hu X, Wang L, Wang X (2014). Reconstruction of the carotenoid biosynthetic pathway of *Cronobacter sakazakii* BAA894 in *Escherichia coli*. PLoS ONE.

[20] McNerney MP, Styczynski MP (2017). Precise control of lycopene production to enable a fast-responding, minimal-equipment biosensor. Metab. Eng..

[21] Lim CG (2015). Development of a recombinant *Escherichia coli* strain for overproduction of the plant pigment anthocyanin. Appl. Environ. Microbiol..

[22] Hui CY (2020). Genetic control of violacein biosynthesis to enable a pigment-based whole-cell lead biosensor. RSC Adv..

[23] Muller M, Auslander S, Auslander D, Kemmer C, Fussenegger M (2012). A novel reporter system for bacterial and mammalian cells based on the non-ribosomal peptide indigoidine. Metab. Eng..

[24] Watstein DM, Styczynski MP (2018). Development of a pigment-based whole-cell zinc biosensor for human serum. ACS Synth. Biol..

[25] Yoshida K (2008). Novel carotenoid-based biosensor for simple visual detection of arsenite: characterization and preliminary evaluation for environmental application. Appl. Environ. Microbiol..

[26] McNerney MP, Michel CL, Kishore K, Standeven J, Styczynski MP (2019). Dynamic and tunable metabolite control for robust minimal-equipment assessment of serum zinc. Nat. Commun..

[27] Wang D (2020). Visual detection of Hg2+ by manipulation of pyocyanin biosynthesis through the Hg2+ -dependent transcriptional activator MerR in microbial cells. J. Biosci. Bioeng..

[28] Joe MH (2012). Pigment-based whole-cell biosensor system for cadmium detection using genetically engineered *Deinococcus radiodurans*. Bioprocess Biosyst. Eng..

[29] Yong YC, Zhong JJ (2009). A genetically engineered whole-cell pigment-based bacterial biosensing system for quantification of N-butyryl homoserine lactone quorum sensing signal. Biosens. Bioelectron..

[30] Cauz ACG (2019). Violacein targets the cytoplasmic membrane of bacteria. ACS Infect Dis..

[31] Choi, S.Y., Yoon, K.H., Lee, J.I. & Mitchell, R.J. Violacein: properties and production of a versatile bacterial pigment. *Biomed Res Int* **2015**, 465056 (2015). 10.1155/2015/465056 PMC4538413 26339614

[32] Morohoshi T, Kato M, Fukamachi K, Kato N, Ikeda T (2008). *N*-acylhomoserine lactone regulates violacein production in *Chromobacterium violaceum* type strain ATCC 12472. FEMS Microbiol. Lett..

[33] Wei H, Cheng H, Ting M, Wen-Hui Z, Xian-Gui L (2010). A chromosomally based luminescent bioassay for mercury detection in red soil of China. Appl. Microbiol. Biotechnol..

[34] Park SJ, Wireman J, Summers AO (1992). Genetic analysis of the Tn21 mer operator-promoter. J. Bacteriol..

[35] Chang CC, Lin LY, Zou XW, Huang CC, Chan NL (2015). Structural basis of the mercury(II)-mediated conformational switching of the dual-function transcriptional regulator MerR. Nucleic Acids Res..

[36] August PR (2000). Sequence analysis and functional characterization of the violacein biosynthetic pathway from *Chromobacterium violaceum*. J. Mol. Microbiol. Biotechnol..

[37] Mathema VB, Thakuri BC, Sillanpaa M (2011). Bacterial *mer* operon-mediated detoxification of mercurial compounds: a short review. Arch. Microbiol..

[38] Priyadarshi H (2012). A GFP-based bacterial biosensor with chromosomally integrated sensing cassette for quantitative detection of Hg(II) in environment. J. Environ. Sci. (China).

[39] Mahbub K, Krishnan K, Naidu R, Megharaj M (2017). Development of a whole cell biosensor for the detection of inorganic mercury. Environ. Technol. Inno.

[40] Selifonova O, Burlage R, Barkay T (1993). Bioluminescent sensors for detection of bioavailable Hg(II) in the environment. Appl. Environ. Microbiol..

[41] Rasmussen LD, Turner RR, Barkay T (1997). Cell-density-dependent sensitivity of a mer-lux bioassay. Appl. Environ. Microbiol..

[42] Zhang NX (2021). Versatile artificial mer operons in *Escherichia coli* towards whole cell biosensing and adsorption of mercury. PLoS ONE.

[43] Zhang NX, Guo Y, Yang XQ, Zhang W, Huang XQ (2018). Surface display of metal binding domain derived from PbrR on *Escherichia coli* specifically increases lead(II) adsorption. Biotechnol. Lett..

[44] Hui C (2018). Surface display of PbrR on *Escherichia coli* and evaluation of the bioavailability of lead associated with engineered cells in mice. Sci. Rep..

[45] Guo M (2019). Using the promoters of MerR family proteins as "rheostats" to engineer whole-cell heavy metal biosensors with adjustable sensitivity. J. Biol. Eng..

[46] Cai S (2018). Engineering highly sensitive whole-cell mercury biosensors based on positive feedback loops from quorum-sensing systems. Analyst.

[47] USEPA. *National Recommended Water Quality Criteria* (1984).

[48] Brown AS, Robins KJ, Ackerley DF (2017). A sensitive single-enzyme assay system using the non-ribosomal peptide synthetase BpsA for measurement of L-glutamine in biological samples. Sci. Rep..

[49] Rodrigues AL (2013). Systems metabolic engineering of *Escherichia coli* for production of the antitumor drugs violacein and deoxyviolacein. Metab. Eng..

[50] Kim H, Lee W, Yoon Y (2019). Heavy metal(loid) biosensor based on split-enhanced green fluorescent protein: development and characterization. Appl. Microbiol. Biotechnol..

[51] Guo M (2020). A test strip platform based on a whole-cell microbial biosensor for simultaneous on-site detection of total inorganic mercury pollutants in cosmetics without the need for predigestion. Biosens. Bioelectron..

[52] Din G (2019). Engineering a bioluminescent bioreporter from an environmentally sourced mercury-resistant Enterobacter cloacae strain for the detection of bioavailable mercury. J. Appl. Microbiol..

