# Supplementary material for: Development of a bioavailable Hg(II) sensing system based on MerR-regulated visual pigment biosynthesis
Source: Sci Rep. 2021 Jun 29;11:13516. doi: 10.1038/s41598-021-92878-6 (PMC8242042; doi:10.1038/s41598-021-92878-6)
Supplement: Supplementary file 1 — Supplementary Information. [file 41598_2021_92878_MOESM1_ESM.pdf]

Supplemental Information

Development of a bioavailable Hg(II) sensing system based on MerR-regulated visual pigment biosynthesis

Yan Guo[1], Chang-ye Hui[2*], Lisa Liu[3], Min-peng Chen[1] & Hong-ying Huang[1]

[1] National Key Clinical Specialty of Occupational Diseases, Shenzhen Prevention and Treatment Center for Occupational Diseases, Shenzhen, China

[2] Department of Pathology & Toxicology, Shenzhen Prevention and Treatment Center for Occupational Diseases, Shenzhen, China

[3] Lewis Katz School of Medicine, Temple University, Pennsylvania, USA

Correspondence and requests for materials should be addressed to C.H. (email: hcy_sypu@hotmail.com)

AGATCTCTAAGGCATAGCTGACCTTGCCAGGCCTGCTTCGCCCTGTAGT
GACGCGATCAACGGGCAGGAAACATTCCCCTTTCGTGCATGGCAGGCG
CACACGAGTTCAGACAGCACGGTTTCCATGCGCGCCAAGTCGGCCATCT
TCTCGCGCACGTCCTTGAGCTTGTGTTCGGCCAGGCTGCTGGCCTCCTC

merR

GCAGTGGGTGCCATCGTCGAGCCGCAACAGCTCGGCAATCTCGTCCAG
ACTGAACCCCAGCCGCTGTGCCGATTTCACGAATTTCACCCGAACCACG
TCCGCCTCCCCATAGCGGCGGATGCTGCCGTAAGGCTTGTCCGGTTCC
CGCAACAGGCCCTTGCGCTGATAGAAGCGGATTGTCTCCACGTTGACCC
CGGCCGCCTTGGCAAAAACGCCAATGGTCAGGTTTTCCAAATTATTTTCC

mer bidirectional promoter region

ATATCGCTTGACTCCGTACATGAGTACGGAAGTAAGGTTACGCTATCCAA
TCCAAATTCAAAAGGGCCAACGT**TCTAGA**AATAATTTTGTTTAACTTTAAG

rbs *NdeI* Violacein expression cassette *SacI*

AAGGAGATATACATATG—***vioA-vioB-vioC-vioD-vioE***—TAA**GAGCTC**CGT
CGACAAGCTTGCGGCCGCACTCGAGCACCACCACCACCACCACTGAGA
TCCGGCTGCTAACAAAGCCCGAAAGGAAGCTGAGTTGGCTGCTGCCAC

T7 terminator

CGCTGAGCAATAACTAGCATAACCCCTTGGGGCCTCTAAACGGGTCTTG
AGGGGTTTTTTG

pPmer-vio

AGATCTCTAAGGCATAGCTGACCTTGCCAGGCCTGCTTCGCCCTGTAGT
GACGCGATCAACGGGCAGGAAACATTCCCCTTTCGTGCATGGCAGGCG
CACACGAGTTCAGACAGCACGGTTTCCATGCGCGCCAAGTCGGCCATCT
TCTCGCGCACGTCCTTGAGCTTGTGTTCGGCCAGGCTGCTGGCCTCCTC

merR

GCAGTGGGTGCCATCGTCGAGCCGCAACAGCTCGGCAATCTCGTCCAG
ACTGAACCCCAGCCGCTGTGCCGATTTCACGAATTTCACCCGAACCACG
TCCGCCTCCCCATAGCGGCGGATGCTGCCGTAAGGCTTGTCCGGTTCC
CGCAACAGGCCCTTGCGCTGATAGAAGCGGATTGTCTCCACGTTGACCC
CGGCCGCCTTGGCAAAAACGCCAATGGTCAGGTTTTCCAAATTATTTTCC

mer bidirectional promoter region

ATATCGCTTGACTCCGTACATGAGTACGGAAGTAAGGTTACGCTATCCAA
TCCAAATTCAAAAGGGCCAACGT**TCTAGA**AATAATTTTGTTTAACTTTAAG

rbs *Nde*I

AAGGAGATATACATATGGTTTCTAAAGGCGAAGAACTGTTCACCGGCGTT
GTTCCGATCCTGGTTGAACTGGATGGTGATGTTAACGGCCACAAATTCAG
CGTCAGCGGCGAAGGCGAAGGCGATGCGACCTACGGCAAACTGACCCT
GAAATTCATCTGCACCACCGGTAAACTGCCGGTTCCGTGGCCGACCCTG
GTTACCACCCTGACCTACGGCGTTCAGTGCTTCAGCCGTTACCCGGATCA
CATGAAACAGCACGATTTCTTCAAAAGCGCGATGCCGGAAGGCTACGTTC
AGGAACGTACCATCTTCTTCAAGGATGATGGCAACTACAAAACCCGTGCG

eGFP

GAAGTTAAATTCGAAGGCGATACGCTGGTTAACCGTATCGAACTGAAAGG
CATCGATTTCAAAGAAGATGGTAACATCCTGGGGCACAAACTGGAATACA
ACTACAACAGCCACAACGTTTATATCATGGCCGACAAACAGAAAAACGGA
ATCAAAGTTAATTTCAAGATTCGCCACAATATCGAAGACGGTTCTGTGCAA
CTTGCAGATCATTACCAGCAAAACACCCCAATTGGCGATGGACCCGTCCT
GCTGCCGGACAACCATTACCTGTCGACACAGTCAGCGCTGTCCAAGGAT
CCGAACGAAAAACGTGATCACATGGTTCTGCTGGAATTCGTTACCGCGGC

*Hind*III

GGGCATCACCCTGGGTATGGATGAACTGTACAAATAA**AAGCTT**GCGGCC
GCACTCGAGCACCACCACCACCACCACTGAGATCCGGCTGCTAACAAAG
CCCGAAAGGAAGCTGAGTTGGCTGCTGCCACCGCTGAGCAATAACTAGC
ATAACCCCTTGGGGCCTCTAAACGGGTCTTGAGGGGTTTTTTG

pPmer-G

AGATCTCTAAGGCATAGCTGACCTTGCCAGGCCTGCTTCGCCCTGTAGT
GACGCGATCAACGGGCAGGAAACATTCCCCTTTCGTGCATGGCAGGCG
CACACGAGTTCAGACAGCACGGTTTCCATGCGCGCCAAGTCGGCCATCT
TCTCGCGCACGTCCTTGAGCTTGTGTTCGGCCAGGCTGCTGGCCTCCTC
merR
GCAGTGGGTGCCATCGTCGAGCCGCAACAGCTCGGCAATCTCGTCCAG
ACTGAACCCCAGCCGCTGTGCCGATTTCACGAATTTCACCCGAACCACG
TCCGCCTCCCCATAGCGGCGGATGCTGCCGTAAGGCTTGTCCGGTTCC
CGCAACAGGCCCTTGCGCTGATAGAAGCGGATTGTCTCCACGTTGACCC
CGGCCGCCTTGGCAAAAACGCCAATGGTCAGGTTTTCCAAATTATTTTCC
mer bidirectional promoter region
ATATCGCTTGACTCCGTACATGAGTACGGAAGTAAGGTTACGCTATCCAA
TCCAAATTCAAAAGGGCCAACGT**TCTAGA**AATAATTTTGTTTAACTTTAAG
rbs *Nde*I Violacein expression cassette *Sac*I
AAGGAGATATACATATG—***vioA-vioB-vioC-vioD-vioE***—TAA**GAGCTC**ATC
GCTTGACTCCGTACATGAGTACGGAAGTAAGGTTACGCTATCCAATCCAA
rbs *Not*I
ATTCAAAAGGGCCAACGTGAAGGAGATATACCATG—*eGFP*—TAA**GCGG**
CCGCACTCGAGCACCACCACCACCACCACTGAGATCCGGCTGCTAACAA
AGCCCGAAAGGAAGCTGAGTTGGCTGCTGCCACCGCTGAGCAATAACTA
GCATAACCCCTTGGGGCCTCTAAACGGGTCTTGAGGGGTTTTTTG

pPmer-vio-Pmer-G

Fig S1. The cloning/expression region of vectors used in this study. DNA sequence and annotation data are all marked.

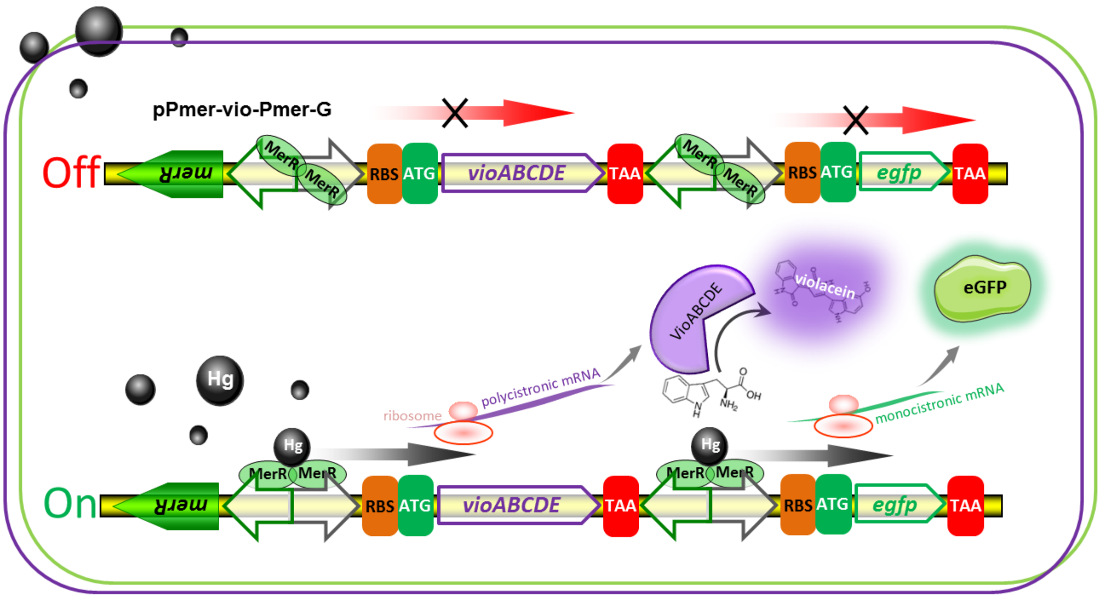

Fig S2. Genetic construct for sensing bioavailable Hg(II) with two reporters. The violacein biosynthesis module and eGFP reporter module were placed under the control of its own *mer* promoter separately in one genetic construct. Upon binding of bioavailable Hg(II) to the dimeric MerR, the protein-metal-ion complex switches from a repressor to an activator to allow the transcription of two reporters.

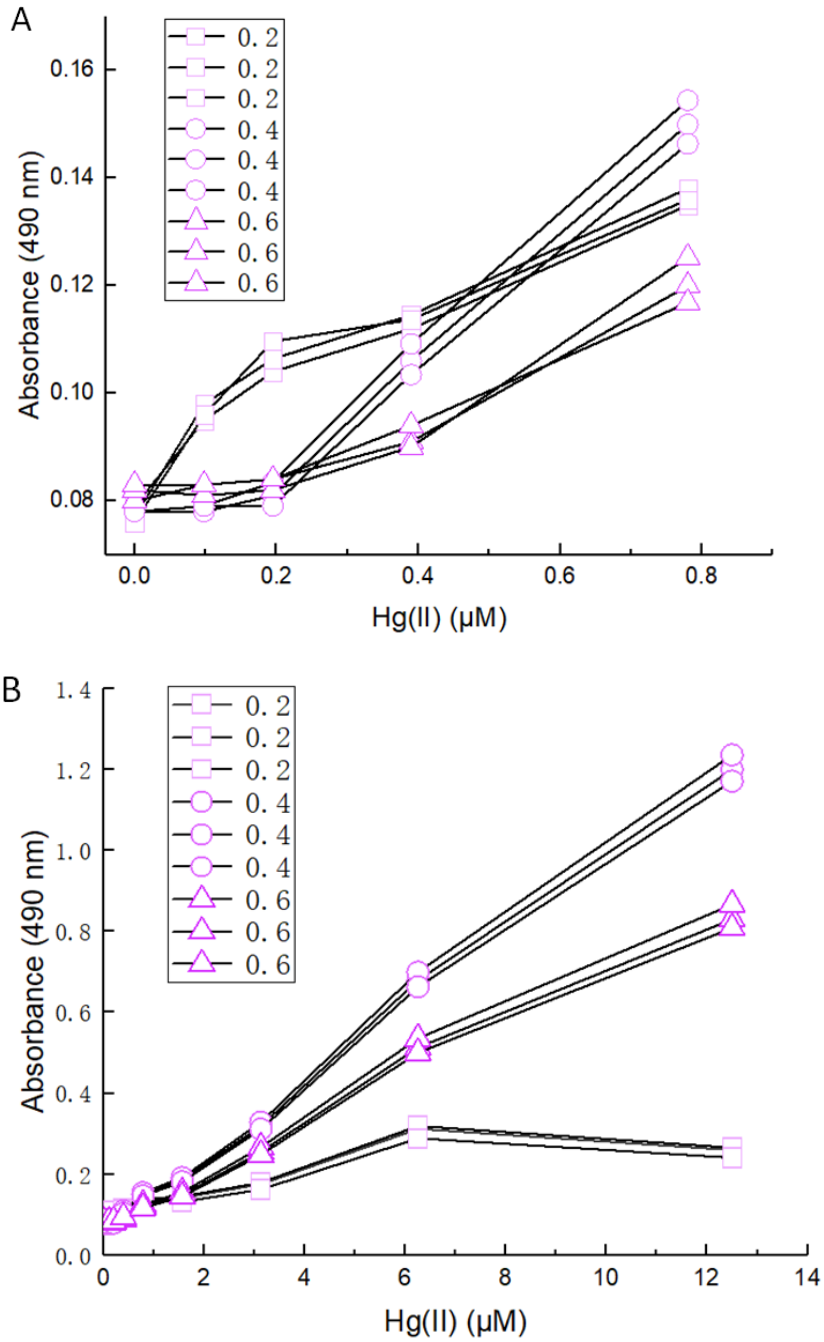

Fig S3. The response of whole-cell biosensor TOP10/pPmer-vio induced with increased concentrations of Hg(II). Early exponential-phase (OD_{600}=0.2), exponential-phase (OD_{600}=0.4), and late exponential-phase (OD_{600}=0.6) cultures (with three repeats) were induced with 0, 0.098, 0.195, 0.39, 0.78, 1.56, 3.125, 6.25, and 12.5 μM Hg(II) at 37 ^{o}C for 12 h. Whole-cell biosensor dose-response curves with Hg(II) concentrations range from 0-0.78 μM (A), and dose-response curves with Hg(II) concentrations range from 0-12.5 μM
